# RR interval variability in the evaluation of ventricular tachycardia and effects of implantable cardioverter defibrillator therapy

**DOI:** 10.1002/joa3.12551

**Published:** 2021-05-18

**Authors:** Keita Tsukahara, Yasushi Oginosawa, Yoshihisa Fujino, Toshihiro Honda, Kan Kikuchi, Masatsugu Nozoe, Takayuki Uchida, Hitoshi Minamiguchi, Koichiro Sonoda, Masahiro Ogawa, Takeshi Ideguchi, Yoshihisa Kizaki, Toshihiro Nakamura, Kageyuki Oba, Satoshi Higa, Keiki Yoshida, Keishiro Yagyu, Taro Miyamoto, Yasunobu Yamagishi, Hisaharu Ohe, Ritsuko Kohno, Masaharu Kataoka, Yutaka Otsuji, Haruhiko Abe

**Affiliations:** ^1^ The Second Department of Internal Medicine University of Occupational and Environment Health Kitakyushu Japan; ^2^ The Department of Environmental Epidemiology Institute of Industrial Ecological Sciences University of Occupational and Environmental Health Kitakyushu Japan; ^3^ Division of Cardiology Kumamoto Junkankika Hospital Kumamoto Japan; ^4^ Division of Cardiology Japan Community Healthcare Organization Kyushu Hospital Kitakyusyu Japan; ^5^ Division of Cardiology Saiseikai Fukuoka General Hospital Fukuoka Japan; ^6^ Department of Cardiovascular Surgery Iizuka Hospital Iizuka Japan; ^7^ Department of Cardiology Osaka Police Hospital Osaka Japan; ^8^ Department of Cardiology Sasebo City General Hospital Sasebo Japan; ^9^ Department of Cardiology Fukuoka University School of Medicine Fukuoka Japan; ^10^ Department of Internal Medicine, Circulatory and Body Fluid Regulation Faculty of Medicine University of Miyazaki Miyazaki Japan; ^11^ Department of Cardiology Sasebo Chuo Hospital Nagasaki Japan; ^12^ Department of Cardiology National Hospital Organization Kyushu Medical Center Fukuoka Japan; ^13^ Department of Cardiology Yuai Medical Center Okinawa Japan; ^14^ Cardiac Electrophysiology and Pacing Laboratory Division of Cardiovascular Medicine Makiminato Central Hospital Okinawa Japan; ^15^ Department of Cardiology Saga‐ken Medical Centre Koseikan Saga Japan; ^16^ Department of Heart Rhythm Management University of Occupational and Environment Health Kitakyusyu Japan

**Keywords:** anti‐tachycardia pacing therapy, implantable cardioverter defibrillator, RR interval variability, shock therapy, ventricular tachycardia

## Abstract

**Background:**

An implantable cardioverter defibrillator (ICD) is the most reliable therapeutic device for preventing sudden cardiac death in patients with sustained ventricular tachycardia (VT). Regarding its effectiveness, targeted VT is defined based on the tachyarrhythmia cycle length. However, variations in RR interval variability of VTs may occur. Few studies have reported on VT characteristics and effects of ICD therapy according to the RR interval variability. We aimed to identify the clinical characteristics of VTs and ICD therapy effects according to the RR interval variability.

**Methods:**

We analyzed 821 VT episodes in 69 patients with ICDs or cardiac resynchronization therapy defibrillators. VTs were classified as irregular when the difference between two successive beats was >20 ms in at least one of 10 RR intervals; otherwise, they were classified as regular. We evaluated successful termination using anti‐tachycardia pacing (ATP)/shock therapy, spontaneous termination, and acceleration between regular and irregular VTs. The RR interval variability reproducibility rates were evaluated.

**Results:**

Regular VT was significantly more successfully terminated than irregular VT by ATP. No significant difference was found in shock therapy or VT acceleration between the regular and irregular VTs. Spontaneous termination occurred significantly more often in irregular than in regular VT cases. The reproducibility rates of RR interval variability in each episode and in all episodes were 89% and 73%, respectively.

**Conclusions:**

ATP therapy showed greater effectiveness for regular than for irregular VT. Spontaneous termination was more common in irregular than in regular VT. RR interval variability of VTs seems to be reproducible.

## Graphical abstract

ATP therapy showed higher effectiveness for regular than for irregular VT. Spontaneous termination of VTs was more common in irregular than in regular VT. RR interval variability of VTs is reproducible.

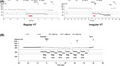

## 1 INTRODUCTION

**FIGURE 1:**
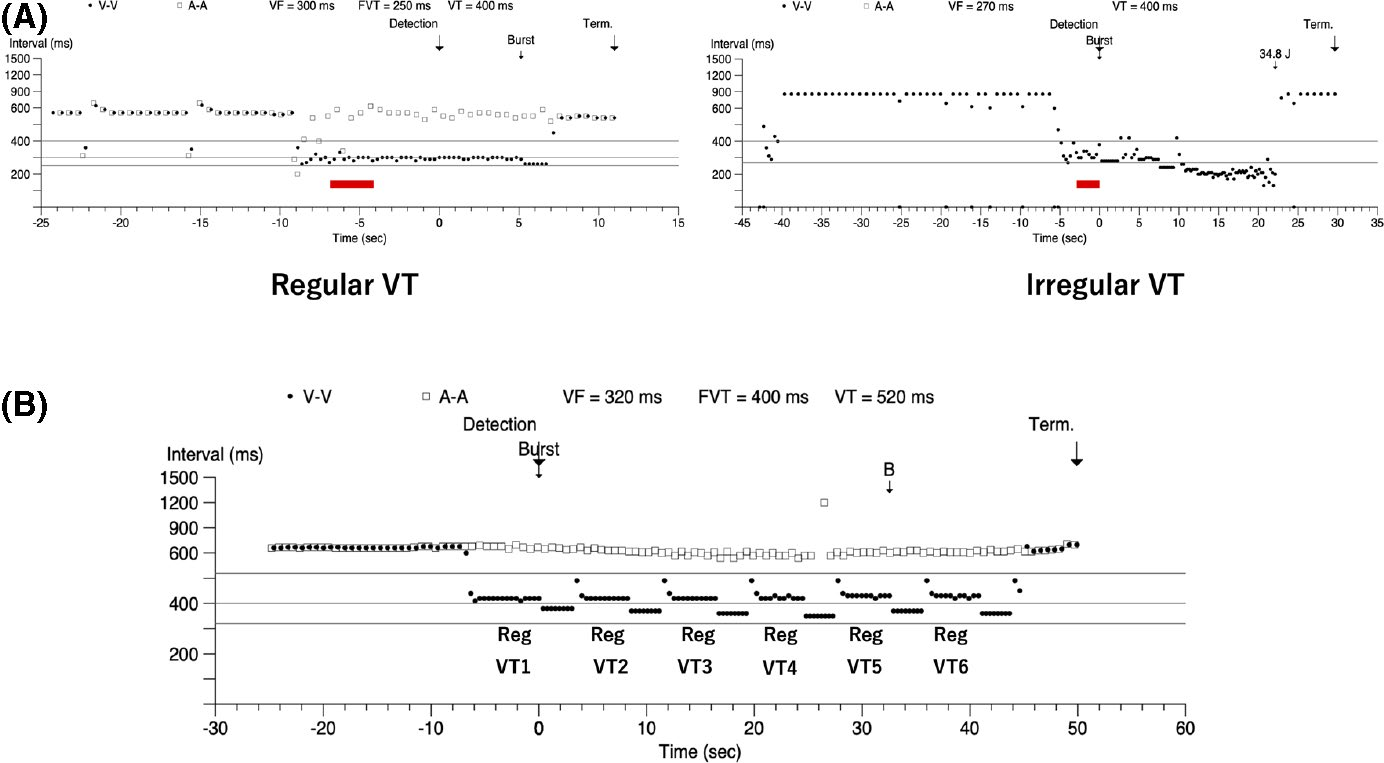
(A) Difference in the cycle length variability of VT. The left panel shows a constant cycle length, whereas the right panel shows the variation in the cycle length. (B) Example of multiple VT episodes within an episode. Regular VTs account for a larger number of VT types, classified by the variability within an episode. The RE value is calculated by dividing the number of regular VTs (6) to the number of total VTs in the episode (6). A‐A, atrial cycle length; RE, reproducibility in each episode; Reg, regular; Term, termination; VF, ventricular fibrillation; VT, ventricular tachycardia; V‐V, ventricular cycle length

An implantable cardioverter defibrillator (ICD) is currently the most reliable therapy for preventing sudden death in patients with sustained ventricular tachycardia (VT) associated with organic heart disease.^1^, ^2^, ^3^, ^4^ ICDs can terminate life‐threatening VTs by using electrical shocks, anti‐tachycardia pacing (ATP), or both. Recent studies have shown that electrical shocks for ventricular tachyarrhythmia may lead to a worse prognosis, such as mortality or morbidity.^5^, ^6^ Therefore, it is essential to reduce the use of shock therapy to terminate VT. Although the effectiveness of ATP therapy has been extensively reported,^7^, ^8^, ^9^ it occasionally induces VT acceleration^10^, ^11^ or progression to ventricular fibrillations (VFs). Many studies on the effectiveness of ICD therapy have reported that the cycle length defines the VTs that are best targeted by ICD therapy. However, in addition to the cycle length, the targeted VTs are characterized by RR interval variability (Figure 1A). Variations in the RR interval in tachyarrhythmia distinguish VTs from supraventricular tachycardia, such as atrial fibrillation.^12^ Concerning the RR interval variability in VT, few studies have reported on the clinical characteristics of VT or the effectiveness of ATP therapy.

This study aimed to evaluate the relationship between RR interval variability in VTs and clinical characteristics of VT, including the effects of ICD therapy.

## 2 METHODS

VTs per episode, recorded using an ICD or a cardiac resynchronization therapy defibrillator (CRT‐D) device, were classified as regular or irregular according to the RR interval variability of the cycle length. Based on this variability, we evaluated the rates of VT termination using ATP or shock therapy, spontaneous termination, and acceleration. Additionally, the reproducibility rates of the RR interval variability of VTs (regular or irregular) in each episode and in all episodes were evaluated.

### 2.1 Study design and population

This was a retrospective, multicenter, observational study. The study protocol was approved by the institutional review board of each participating center. Data analyzed were collected from 69 patients who experienced episodes of ventricular arrhythmias. The participants were selected among 185 patients involved in the Defibrillator with Enhanced Features and Settings for Reduction of Inaccurate Detection (DEFENSE) Trial.^13^ Briefly, that study compared the SmartShock Technology (SST) algorithm with the conventional VT detection algorithms. The trial enrolled consecutive recipients of an ICD or CRT‐D that used the SST algorithm (Protecta XT ICD [DR, VR], Protecta XT CRT‐D [DR], and Evera XT ICD [DR, VR]; Medtronic, Minneapolis, MN, USA). Patients whose device programing did not match the study requirements (Table S1) were unable to complete a 2‐year follow‐up period, or were unable to provide informed consent were excluded from this research. The recipients were followed up every 6 mo for up to 2 y after device implantation, by remote monitoring of their device or by presenting in the outpatient clinic. The devices were assessed at all scheduled and unscheduled follow‐up visits. The DEFENSE trial revealed that, compared with the conventional algorithms, the SST discrimination algorithm significantly lowered the rate of inaccurate VT detection.

VT episodes were evaluated as follows: of the 185 patients initially enrolled, 69 experienced 821 episodes of ventricular arrhythmias (VT, fast VT, or VF); of the 821 episodes, 608 (74%) and 213 (26%) were judged as true ventricular tachyarrhythmia and as other episodes, respectively, including atrial fibrillation/atrial flutter (n = 26), sinus tachycardia or atrial tachycardia (AT) (n = 178), and T‐wave oversensing (n = 9), leaving data from just 53 patients who exhibited true ventricular tachyarrhythmia. Each entire episode was reviewed by an independent adjudication committee to determine whether the diagnosis was appropriate.

### 2.2 Analysis of the RR interval variability of VT

The DEFENSE trial guidelines recommended that the devices should have been programed to provide therapy for VTs with a cycle length <400 ms (Table S1). Of the aforementioned 608 true VT/VF episodes, only cycle lengths ≥240 ms were considered as VT episodes in this study.

VT cycle length variability was determined based on a previous report; 10 RR intervals, from 2 s after the onset of VT on intracardiac electrograms, were obtained by automatic reading of the devices, with an accuracy of 10 ms^12^ VT was judged as irregular when any difference between two successive beats was >20 ms at least once in 10 RR intervals; otherwise, it was classified as regular.^12^ When ATP was administered more than once in an episode, evaluation of the RR interval variability after the first therapy session was performed using the 10 RR intervals occurring immediately before any subsequent ATP therapy.

### 2.3 VT termination

Episode termination was used to confirm the classification of success by ATP therapy. Termination occurring more than five beats after therapy was deemed unsuccessful and was classified as spontaneous termination after ATP delivery.^10^ Furthermore, VTs that terminated naturally without ATP or shock therapy were defined as spontaneous termination without therapy.

### 2.4 VT acceleration

Ventricular rhythm acceleration following ATP therapy was defined as a 10% decrease in the cycle length.^10^

### 2.5 Reproducibility of the RR interval variability of VT

We examined the reproducibility of the RR interval variability of VT from two perspectives: reproducibility in each episode (RE) and reproducibility in all episodes (RA).

RE was evaluated when there were two or more VTs in one episode (ie, ATP therapy was performed more than once in an episode). Of the VTs classified on the basis of the RR interval variability within an episode, the ratio of the larger number of VTs by type (ie, regular or irregular) was defined as RE (Figure 1B). When the numbers of regular and irregular VTs were identical in an episode, the episode was assigned according to the variability of the first recorded VT in the episode.

**FIGURE 2:**
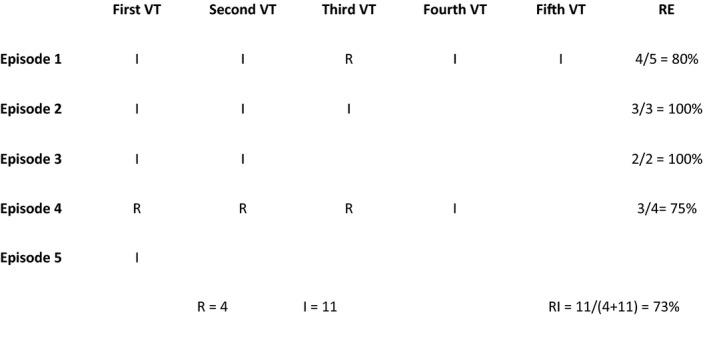
A patient with five VT episodes. Most VTs in all episodes are irregular. The RI value is calculated by dividing the number of irregular VTs in all episodes of the patient (11) by the number of total VT episodes (15), ie, 11/15 = 0.73. I, irregular VT; R, regular VT; RA, reproducibility in all episodes; VT, ventricular tachycardia

RA was assessed in patients with multiple VT episodes. The ratio of the larger number of VTs by type to the number of total VT episodes in all episodes within an individual was defined as RA (Figure 2).

### 2.6 Statistical analysis

Continuous data are presented as means and standard deviations. Univariate and multivariate odds ratios (ORs) for VT termination and acceleration were estimated. A multilevel logistic regression was applied to estimate the ORs as VT episodes were nested within individuals. The multivariate model included the RR interval variability of VTs (regular or irregular), average VT rate (beats per min [bpm]), sex, ischemic cardiomyopathy, and use of an antiarrhythmic drug, β‐blocker, angiotensin‐converting enzyme inhibitor (ACE‐I), or angiotensin II receptor blocker (ARB). Statistical analysis was performed using STATA version 16 (StataCorp LP, College Station, TX, USA). The level of statistical significance was set at *P* < .05.

## 3 RESULTS

### 3.1 Study groups

**FIGURE 3:**
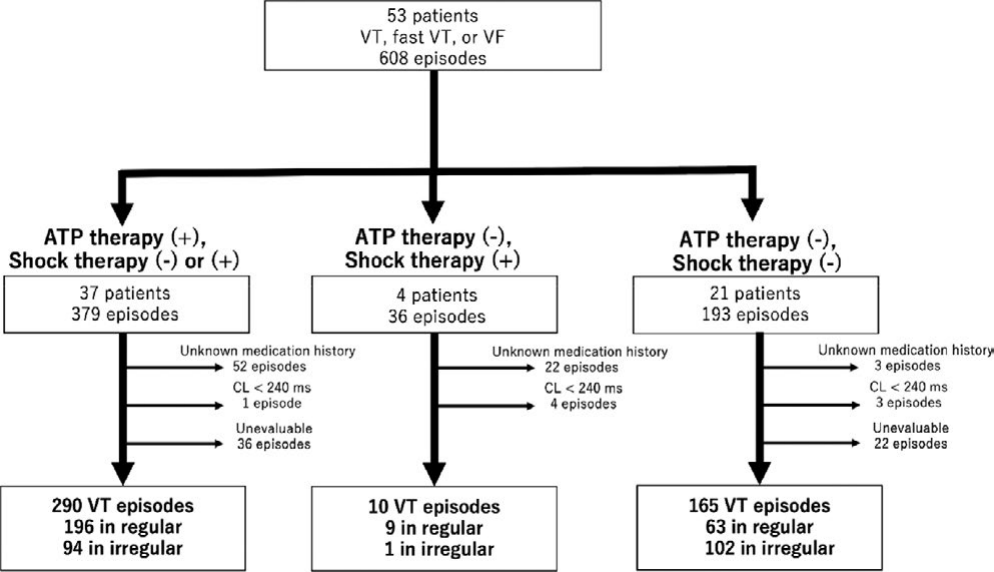
Flow chart of patient selection. ATP, anti‐tachycardia pacing; CL, cycle length; VF, ventricular fibrillation; VT, ventricular tachycardia

In a study group of 53 patients with an ICD or CRT‐D, cases of episodes with an unknown medication history (n = 77 in seven patients), a cycle length <240 ms (n = 8 in seven patients), and a starting point of ventricular tachyarrhythmia that could not be confirmed on intracardiac electrocardiogram (n = 58 in seven patients) were excluded. Consequently, the remaining 465 episodes in 43 patients were included in the present analyses (Figure 3, Tables 1 and 2).

**TABLE 1:** Characteristics of patients with episodes of ventricular tachycardia

Number of patients	n = 43
Male sex	36 (84%)
Ischemic cardiomyopathy	20 (47%)
Non‐ischemic cardiomyopathy (DCM/sarcoidosis/HCM/amyloidosis/Brugada S/unclassified)	23 (53%) (10/3/2/1/1/6)
NYHA class (I/II/III/IV)	(12/19/11/1)
ß‐blocker use	31 (72%)
ACE‐I or ARB use	28 (65%)
Cardiotonic agent use	7 (16%)
Antiarrhythmic drug use	13 (30%)
EF (%)	36 ± 15
CRT‐D	16 (37%)

Note

Data are presented as numbers, unless otherwise indicated.

Abbreviated: ACE‐I, angiotensin‐converting enzyme inhibitor; ARB, angiotensin II receptor blocker; Brugada S, Brugada syndrome; CRT‐D, cardiac resynchronization therapy defibrillator; DCM, dilated cardiomyopathy; EF, ejection fraction; HCM, hypertrophic cardiomyopathy; NYHA, New York Heart Association

**TABLE 2:** Relationship between patient background and RR interval variability of VT

	Regular VT (n = 268)	Irregular VT (n = 197)
Average VT rate (bpm)	178 ± 26	178 ± 22
Male sex	218 (81%)	176 (89%)
Ischemic cardiomyopathy	119 (44%)	75 (38%)
Antiarrhythmic drug use	74 (28%)	49 (25%)
ß‐blocker use	198 (74%)	149 (76%)
ACE‐I or ARB use	169 (63%)	149 (76%)

Note

Data are presented as numbers (%), unless otherwise indicated.

Abbreviated: ACE‐I, angiotensin‐converting enzyme inhibitor; ARB, angiotensin II receptor blocker; VT, ventricular tachycardia; bpm, beats per min

### 3.2 VT termination

Among the 465 episodes in 43 patients, 290 were managed with ATP therapy, 10 with shock therapy without ATP (shock therapy was administered after ATP therapy in 17 episodes), and 165 without therapy.

The characteristics of ATP therapy are summarized in Table S2. The first pacing program for ATP therapy was set as burst pacing in all cases. ATP therapy terminated 85% of VT episodes (n = 246) (regular VT, 94%; irregular VT, 66%), and shock therapy terminated 100% of such episodes (n = 27). After ATP delivery, the rate of spontaneous termination was 5% (n = 24), and the spontaneous termination rate without therapy was 31% (n = 145). VT termination could not be confirmed using intracardiac electrocardiography (ie, episodes that were out of the VT zone without termination) in 5% of the episodes (n = 23).

Concerning the ATP therapy, regular VTs showed significantly more successful terminations than irregular VTs (*P* < .001; OR, 7.56). No significant difference was found in VT termination using ATP therapy between ischemic and non‐ischemic cardiomyopathies. In addition, VT episodes with a faster rate showed a lower termination rate (*P* = .002; OR, 0.97; Table 3). Spontaneous termination after ATP delivery occurred significantly more frequently in irregular than in regular VTs (*P* < .001; OR, 30.58; Table 4). The VT rate had no significant effect on spontaneous termination after ATP delivery. For episodes without therapy, spontaneous termination was more commonly observed in irregular than in regular VTs (*P* = .001; OR, 6.06).

**TABLE 3:** Odds ratios of clinical characteristics for termination using ATP therapy

	n	% of termination using ATP therapy	Univariate analysis			*p*‐value	Multivariate analysis			*p‐*value
			Odds ratio	95% confidence interval			Odds ratio	95% confidence interval		
RR interval variability of VT										
Irregular	94	66%	reference				reference			
Regular	196	94%	5.94	2.50	14.13	< 0.001	7.56	3.05	18.78	< 0.001
Average VT rate (bpm)	290	85%	0.97	0.95	0.99	0.006	0.97	0.95	0.99	0.002
Sex										
Women	71	86%	reference				reference			
Men	219	84%	0.59	0.08	4.40	0.609	0.34	0.06	2.05	0.241
Structural heart disease										
Non‐ischemic cardiomyopathy	124	84%	reference				reference			
Ischemic cardiomyopathy	166	86%	0.63	0.12	3.45	0.597	1.28	0.24	6.94	0.775
Antiarrhythmic drug use										
No	169	88%	reference				reference			
Yes	121	80%	0.86	0.15	4.99	0.864	0.57	0.13	2.63	0.474
β‐blocker use										
No	86	91%	reference				reference			
Yes	204	82%	0.51	0.07	3.48	0.491	0.25	0.04	1.49	0.128
ACE‐I or ARB use										
No	101	85%	reference				reference			
Yes	189	85%	1.10	0.20	6.12	0.914	1.14	0.20	6.43	0.878

Abbreviated: ACE‐I, angiotensin‐converting enzyme inhibitor; ARB, angiotensin II receptor blocker; ATP, anti‐tachycardia pacing; bpm, beats per min; VT, ventricular tachycardia

**TABLE 4:** Odds ratios of clinical characteristics for spontaneous termination after ATP delivery

	n	Spontaneous termination after ATP delivery	Univariate analysis			*p*‐value	Multivariate analysis			*p‐*value
			Odds ratio	95% confidence interval			Odds ratio	95% confidence interval		
RR interval variability of VT										
Irregular	94	23%	reference				reference			
Regular	196	1%	0.03	0.01	0.17	< 0.001	0.03	0.01	0.16	< 0.001
Average VT rate (bpm)	290	85%	0.99	0.97	1.02	0.662	1.00	0.98	1.03	0.701
Sex										
Women	71	7%	reference				reference			
Man	219	9%	1.42	0.15	13.60	0.609	1.81	0.26	12.82	0.550
Structural heart disease										
Non‐ischemic cardiomyopathy	124	9%	reference				reference			
Ischemic cardiomyopathy	166	8%	1.70	0.25	11.67	0.590	0.83	0.12	5.99	0.855
Antiarrhythmic drug use										
No	169	7%	reference				reference			
Yes	121	10%	1.05	0.15	7.48	0.958	0.91	0.17	4.96	0.914
β‐blocker use										
No	86	7%	reference				reference			
Yes	204	9%	1.55	0.17	13.75	0.694	1.86	0.25	14.10	0.548
ACE‐I or ARB use										
No	101	7%	reference				reference			
Yes	189	9%	1.01	0.14	7.05	0.993	0.96	0.13	6.84	0.967

Abbreviated: ACE‐I, angiotensin‐converting enzyme inhibitor; ARB, angiotensin II receptor blocker; ATP, anti‐tachycardia pacing; bpm, beats per min; VT, ventricular tachycardia

### 3.3 VT Acceleration

In 290 VT episodes of ATP therapies, occurrences of VT acceleration were significantly associated with faster than with slower VTs (*P* = .006; OR, 1.04; average VT heart rate of episodes including acceleration vs. no acceleration, 200 ± 21 vs. 178 ± 25 bpm). By contrast, no significant difference was observed in acceleration occurrence in other items (Table 5).

**TABLE 5:** Odds ratios of clinical characteristics for acceleration of VTs

	n	% of Acceleration of VTs	Univariate analysis			*p*‐value	Multivariate analysis			*p‐*value
			Odds ratio	95% confidence interval			Odds ratio	95% confidence interval		
RR interval variability of VT										
Irregular	94	7%	reference				reference			
Regular	196	5%	1.09	0.32	3.79	0.887	1.13	0.32	4.00	0.851
Average VT rate (bpm)	290	85%	1.04	1.01	1.06	0.008	1.04	1.01	1.07	0.006
Sex										
Women	71	4%	reference				reference			
Man	219	6%	1.22	0.10	15.16	0.879	2.64	0.26	27.26	0.416
Structural heart disease										
Non‐ischemic cardiomyopathy	124	6%	reference				reference			
Ischemic cardiomyopathy	166	5%	1.39	0.16	12.07	0.765	1.48	0.11	19.62	0.765
Antiarrhythmic drug use										
No	169	4%	reference				reference			
Yes	121	7%	1.45	0.16	12.94	0.738	1.96	0.28	13.60	0.495
β‐blocker use										
No	86	2%	reference				reference			
Yes	204	7%	3.03	0.24	38.59	0.393	7.17	0.65	79.48	0.109
ACE‐I or ARB use										
No	101	7%	reference				reference			
Yes	189	5%	0.55	0.06	4.74	0.590	0.27	0.02	3.62	0.320

Abbreviated: ACE‐I, angiotensin‐converting enzyme inhibitor; ARB, angiotensin II receptor blocker; bpm, beats per min; VT, ventricular tachycardia

### 3.4 Reproducibility of the RR interval variability of VT

Overall, in 47 episodes from 14 patients, ATP therapy was administered more than once per episode. Of those, 30 and 17 episodes presented a higher proportion of regular and irregular VTs, respectively (12 and eight patients, respectively). Six of these patients had regular and irregular VT episodes. The reproducibility of VT variability within an episode was 89 ± 19% (regular VT, 94 ± 15%; irregular VT, 80 ± 21%). Additionally, 27 patients had more than two VT episodes. The reproducibility of the RR interval variability of VT in individuals was 73 ± 18% (ischemic vs. non‐ischemic, 71 ± 17 vs. 75 ± 18%).

## 4 DISCUSSION

Based on the variability of the VT cycle length, the three important findings of this study were as follows: first, ATP therapy produced a significantly higher termination rate in regular than in irregular VTs. Second, spontaneous termination after ATP delivery or without therapy occurred significantly more frequently in irregular than in regular VT cases. Third, reproducibility of the RR interval variability of VT was high, both per episode and in all episodes.

The mechanism and properties of tachycardia, substrate, local electrophysiology, and stimulation site affect the success of ATP attempts in terminating VTs.^14^ Although it may be difficult to capture all of this information in an ICD, focusing on RR interval variability may be important to improve the effectiveness of ATP treatment. Three types of VT mechanisms are known to date—reentry, triggered activity, and automaticity. In general, regular heart rates are found in VT cases because of the reentry mechanism, and the irregular heart rate is a finding of non‐reentrant VT.^15^

According to previous reports, small changes in the VT cycle length suggested the increase in ATP effectiveness.^16^ That evaluation used the percentage of variation, which was calculated by dividing the mean difference between each RR interval and the next one by the VT cycle length. Furthermore, ATP therapy is more effective for VTs that have smaller ventricular beat‐to‐beat morphologic variation on intracardiac recordings than for those that do not.^17^ We evaluated the VT characteristics using a simple method, different from the one used in previous studies, to simply measure the variability of the VT cycle length.

In addition, ventricular tachyarrhythmia caused by a triggered activity or automaticity of the mechanism can be difficult to terminate using programed electrical stimulation with reproducibility. By contrast, VT caused by the reentrant mechanism can result in successful termination using programed stimulation, without excluding triggered activity.^18^ The efficacy of ATP in these VTs may be explained by the fact that the VT mechanisms were based on reentry and that VTs that demonstrate poor response to ATP therapy could result from the lack of organized reentry.

### 4.1 VT termination

In our study, the successful VT termination rate using ATP was 85%, which is equivalent to those reported in previous studies.^19^, ^20^ In cases of faster VT, the rate was lower, as faster VT has a shorter excitable gap in the reentrant circuit; hence, it is more difficult for the pacing stimulus to enter the circuit.^21^ VT classification, based on the variability of the VT cycle length, revealed that the successful termination rates following ATP therapy were 94% and 66% in regular and irregular VTs, respectively, showing a statistically significant difference. We observed that VTs with a stable cycle length variability are more likely to respond to ATP therapy.

Spontaneous termination after ATP delivery or without therapy was found significantly more often in irregular than in regular VTs. Spontaneous termination after ATP delivery can include purely spontaneous termination and termination because of overdrive pacing, which is a characteristic finding of non‐reentrant mechanisms.^22^

Furthermore, no significant differences were found between patients with ischemic and non‐ischemic cardiomyopathies with respect to VT termination using ATP therapy. Scar‐related reentry is the most common cause of sustained VT in structural heart disease.^23^ In patients with structural heart disease, myocardial infarction is most commonly associated with a damaged myocardium, which serves as a substrate for reentrant arrhythmias. However, scar‐related VT also develops in other myocardial diseases, including dilated cardiomyopathy, sarcoidosis, and arrhythmogenic right ventricular cardiomyopathy, and after cardiac surgery for congenital heart disease or valve replacement.^17^ The scar slows conduction and increases susceptibility to reentrant arrhythmias.

### 4.2 Clinical implication of the RR interval variability of VT on ICD management

As VT regularity is highly reproducible in each episode or in all episodes, it could be possible to construct more effective ICD settings according to the RR interval variability of VT (ie, considering aggressive ATP therapy for regular VTs and extending the VT detection time and initiation of therapy for irregular VTs). In addition, it could be possible to develop a more effective algorithm based on the variability of the RR interval. Further research is needed for this purpose.

### 4.3 Limitations

Our study has several limitations. First, the number of VT episodes per individual varied. Multilevel logistic regression was applied to estimate the ORs, as VT episodes were nested within individuals. Second, the VT evaluation period was limited to 10 RR intervals of 2 s after the onset, to simplify the clinical usage. In some episodes, RR interval variations may have changed after the assessment. Third, we could not perform multivariate analyses for spontaneous termination without therapy, as the number of target episodes was small. Fourth, these results may not be indicative for slow VT cases because the programing of VT detection for each monitor and therapy zone was set at approximately 150 bpm in most episodes.

## 5 CONCLUSIONS

ATP therapy for VT termination is more effective for regular than for irregular VT cases, as determined by the VT cycle length variability. Additionally, irregular VT has a higher rate of spontaneous termination than regular VT, and the VT cycle length variability is reproducible for an episode and an individual.

## CONFLICT OF INTEREST

This study was financially supported by Medtronic Japan. Dr Satoshi Higa is a consultant to Japan Life Line and Johnson & Johnson and received speaker's honoraria from Japan Life Line, Medtronic, Abbott, Bayer, Biotronik, Boehringer‐Ingelheim, Bristol‐Myers, Daiichi‐Sankyo Pharmaceutical Company, and Pfizer.

## ETHICAL APPROVAL

The study protocol was approved by the institutional review board of each participating center. Written informed consent was obtained from the patients for obtaining the data.

## Supporting information

Supplementary Material

## Data Availability

The study data are available upon request.
