# Supplementary material for: RR interval variability in the evaluation of ventricular tachycardia and effects of implantable cardioverter defibrillator therapy
Source: J Arrhythm. 2021 May 18;37(4):1052–60. doi: 10.1002/joa3.12551 (PMC8339111; doi:10.1002/joa3.12551)
Supplement: Supplementary file 1 — Supplementary Material [file JOA3-37-1052-s001.docx]

**Supplementary Table 1** Recommended program

|  | **Status** | **Criteria** | **Therapy** |
| --- | --- | --- | --- |
| **Zone** |  |  |  |
| Ventricular fibrillation | On | Cycle length; no. of intervals to detection | Physician discretion |
| Ventricular tachycardia | On | Cycle length < 400 ms; | Physician discretion |
|  |  | No. of intervals to |  |
|  |  | detection: 16 |  |
|  |  | Re-detection: 12 |  |
| **SVT limit zone** |  | Physician discretion |  |
| **PR-Logic** | On |  |  |
| **Wavelet** | On or monitor |  |  |
| **Discriminator** |  |  |  |
| T-wave oversensing | On |  |  |
| Lead noise | On or on + timeout |  |  |

**Supplementary Table 2** Characteristics of ATP therapy

|  | **All VT episodes** | **Regular VT** | **Irregular VT** |
| --- | --- | --- | --- |
| **Episodes of ATP therapy** | 290 | 196 (68%) | 94 (32%) |
| **Initial R-S1 interval (%RR) (91/88/84/81)** | (12/221/14/43) | (6/145/8/37) | (6/76/6/6) |
| **Initial pulses (4/6/8/12/14)** | (1/3/231/52/3) | (1/3/147/45/0) | (0/0/84/7/3) |
| **Included ramp pacing** | 5 | 3 (2%) | 2 (2%) |
| **Average VT rate targeted by ATP therapy (bpm)** | 179 | 179 | 181 |
| **ATP times for termination of VT without shock therapy** | 1.35 times | 1.35 times | 1.33 times |
| **Termination by ATP therapy** | 246 (85%) | 184 (94%) | 62 (66%) |
| **Acceleration after ATP therapy** | 16 | 9 (5%) | 7 (7%) |
| **Uncaptured VT episodes by ATP (burst/ramp)** | (5/1) | (4/0) | (1/1) |

Data are presented as n, unless otherwise indicated.

ATP, anti-tachycardia pacing; bpm, beats per min; VT, ventricular tachycardia.
